# Be general, but be specific—hidden traps of model transfer in cell biology

**DOI:** 10.1007/s00709-020-01563-4

**Published:** 2020-10-08

**Authors:** Peter Nick

**Affiliations:** Botanical Institute, Karlsruher Institut für Technologie, Karlsruhe, Germany

Beginning with the time when Theodor Schwann and Matthias Jakob Schleiden (1838) proposed the Cell Theory, cell biology had been one of the most integrative disciplines of biology. Rather than shifting the particularities of life forms into the focus, cell biology strived to reveal their common ground, as different as these life forms might appear at first sight. This general approach paved the way for other disciplines such as evolutionary theory, biochemistry, or molecular biology that helped us to unify our view on living organisms (including ourselves).

A central tool for this unified biology is model organisms that serve as representatives for others, because they are easier to access for observation or manipulation. This tool was so powerful and, especially since the rapid development of molecular methodology, model organisms have gained tremendous analytical power. The undisputed importance of models such as yeast, thale cress, fruit fly, or mouse for a mechanistic understanding of biology comes with its own specific pitfalls, though. The transfer of mechanistic hypotheses from a model organism to those organisms, it stands for, requires a careful consideration of transferability. One of the three criteria for a model identified by the science philosopher Herbert Stachowiak (1973) is reduction (*Verkürzung*), meaning that a model represents those aspects of the original that appear relevant in the context of the question. So, connected tubes are a very good model to understand the physics of blood circulation or to predict vessel diameters using the Hagen-Poiseuille Law, but they do not suffice, if one tries to understand the effect of the parasympathic neural system upon blood pressure, simply, because this aspect has been lost during the reduction of the real-world blood system to a system of tubes (Nick and Gutmann 2019).

Inappropriate reductions are more common than one would expect. They are misleading, because they not only ignore differences that are important to understand particular life forms but they also stimulate the design of experiments that ask into the wrong direction and therefore miss the point. In our days, models determine the allocation of research funds, concepts prevailing in a research community, and even the establishment of entire labs. Thus, if inappropriate reductions are recognised and spelled out, this is usually not fostering harmony, because ideas that have been shared by entire research communities are questioned or even dismissed. However, science lives on a professional handling of errors in the first place. Therefore, it is important for the sake of science as a common human project that errors are named and discussed.

The contribution by Ivanov and Robinson (2020) in the current issue represents such a case, where a misconception deriving from inappropriate model reduction is named, analysed, and corrected. Originally intended as comment to the work by Delgadillo et al. (2020), this review critically questions current concepts of the retromer, a protein complex involved in the recycling of membrane receptors to the trans-Golgi network. Receptor recycling is also known from plants. A classic example is the receptor for bacterial flagellin, an important trigger for basal immunity (Robatzek et al. 2006). Also for the three vacuolar sorting proteins that represent the core of the retromer, homologues exist, as well as for their associates, the sorting nexins. The congruence in functional context and molecular repertory stimulated the notion that the mammalian model for the retromer, the mannosyl 6-phosphate receptor, can be transferred to plants as well. The motivation of the authors for this minireview was to challenge this model transfer from mammalian to plant cells. They show, based on published record, that several implications deriving from this model transfer are not congruent with the empirical evidence. After de-construction of the over-stretched model transfer, they come up with a new improved model that considers the specificity of plant membrane flow.

The main argument for this rectification was the deviating localisation of the vacuolar sorting receptors that are not found at the trans-Golgi network as in the mammalian or the yeast models, but at the multivesicular body. Moreover, treatment with Brefeldin A or Wortmannin, inhibitors of membrane flow that repartition vacuolar sorting proteins, does not alter the localisation of the plant vacuolar sorting proteins. The argument of differing localisation is backed up by the fact that other implications from the mammalian retromer model have not been addressed experimentally. For instance, the physical interaction between the nexins and the bona-fide vacuolar sorting proteins would need to be tested biochemically in vitro (for instance by pull-downs) or by fluorescence-based approaches in situ (for instance by bimodal fluorescence complementation). Furthermore, immunogold labelling assigns the retromer complex to the membrane of the multivesicular body, while the sorting nexins are localised at the trans-Golgi network (Stierhof et al. 2013).

Thus, while all the molecular components seem to be present in plants as well, their subcellular localisation and their functional context recombine differently from the mammalian and yeast models. To simply infer details from these models to the situation in plants can, therefore, be misleading. As long as this inference is conducted explicitly, there is at least the chance to discuss about the pros and cons. However, in most cases, model transfer remains implicit, and then it is much harder to see the hidden traps. It was these implicit conjectures that stimulated the authors to write their minireview. Delgadillo et al. (2020) had discovered that specific vacuolar sorting proteins associated with the microtubular cytoskeleton and proposed that this newly discovered ER- and microtubule-associated compartment (EMAC) would act as sink for retromer vesicles transporting vacuolar sorting receptors from the trans-Golgi network. This working hypothesis was developed based on the information available for the mammalian retromer, but conflicts with the specific differences of the plant retromer mentioned above. The minireview by Ivanov and Robinson (2020) makes this model transfer explicit and spells out, where it is not appropriate.

What do we learn from this interesting case study beyond the particularities of plant vesicle traffic? Nature is a notorious tinkerer (Jacob 1977). This means on the molecular level that the very same proteins can be recruited for different functions. A model transfer that is mainly based on sequence homology can turn into a slippery endeavour, therefore. It is crucial to probe whether localisation and functional context are preserved.

Model organisms emphasise the general aspects, the commonalities. This has been a very powerful strategy. However, during model transfer, it is essential to keep track of the specificities of different life forms. Be general, but be specific.
